# Cystic tumour of the atrioventricular node: case report and literature review

**DOI:** 10.1080/20961790.2019.1595349

**Published:** 2019-05-16

**Authors:** Stephen D. Cohle

**Affiliations:** Spectrum Health, Grand Rapids, MI, USA

**Keywords:** Forensic sciences, forensic pathology, cystic tumour of the AV node, AV node tumour, sudden cardiac death, cardiac arrhythmias

## Abstract

Cystic tumour of the atrioventricular node is the smallest tumour that can cause sudden cardiac death. This lesion arises from foregut endodermal rests which become enfolded into the heart during embryogenesis. Typically causing heart block, the tumour can cause sudden death despite pacemaker placement. Sudden death in such cases can be caused by arrhythmogenic ectopic foci arising from impaired electrical impulse propagation through the abnormal atrioventricular junction conducting tissue.

## Introduction

Sudden cardiac death (SCD) is defined as an unexpected death without an obvious noncardiac cause that occurs within 1 h of witnessed symptom onset or within 24 h of unwitnessed symptom onset. SCD is a major health problem, accounting for an incidence of 69/100 000/year in the United States [1]. Some autopsies of individuals suffering SCD are negative, prompting examination of the cardiac conducting system. Conduction system disease accounted for 30/769 cases (3.9%) of SCD in Ding et al.’s series [2]. Such examination occasionally reveals a cystic tumour of the atrioventricular node (CTAVN), which has been termed the smallest tumour capable of causing sudden death [3]. Of 11 cases of benign cardiac tumours causing sudden death in Patel et al.’s series [4] of ∼1 600 cases, four were CTAVN.

## Case history

The patient was a 45-year-old white female who was found dead on a couch. She had a history of “congenital symptomatic bradycardia” and complete heart block. She had undergone pacemaker placement as child and this was replaced by a second pacemaker a few months before death.

**Figure 1.**
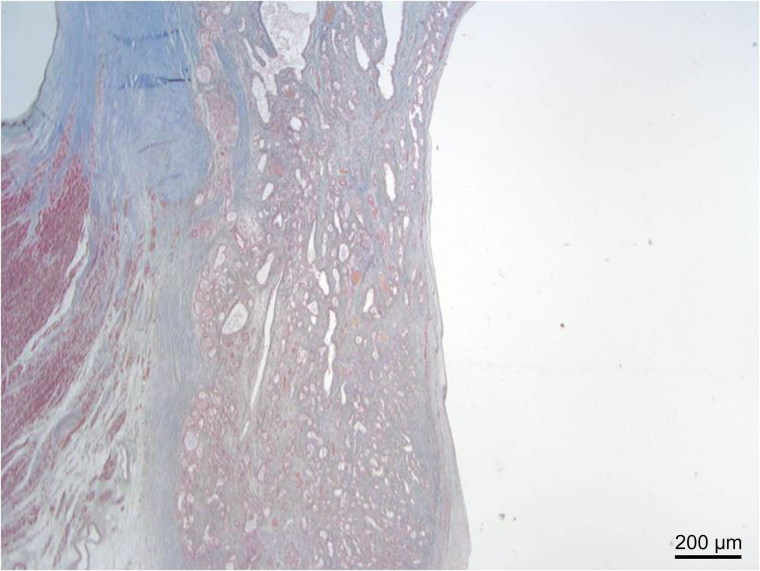
Low power view showing replacement of atrioventricular node by cystic tumour of the atrioventricular node. (Masson trichrome, ×40).

**Figure 2.**
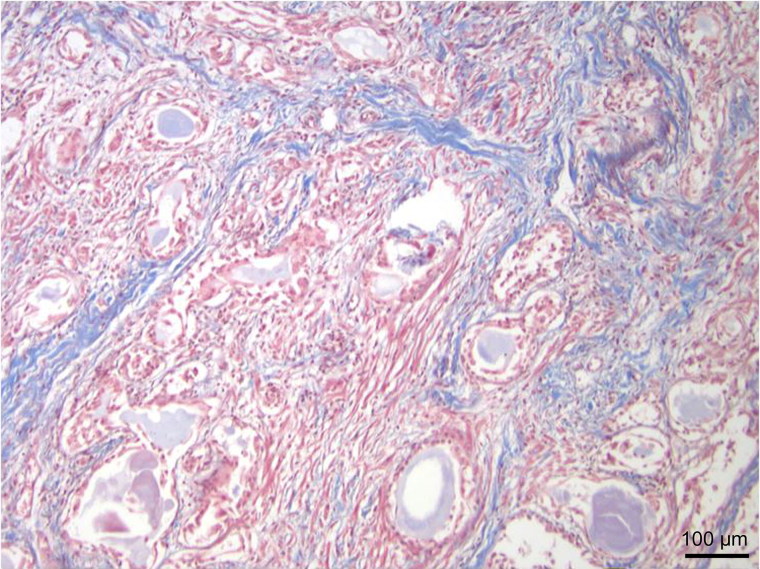
Higher power view showing that the tumour consists of solid nests of cells as well as glands (Masson trichrome, ×200).

**Figure 3.**
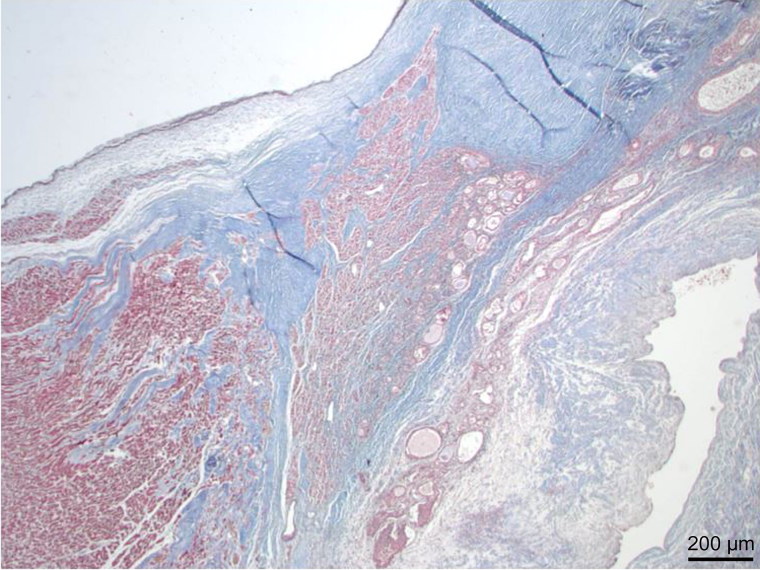
Low power view of His bundle showing partial replacement by glandular elements of tumour (Masson trichrome, ×40).

**Figure 4.**
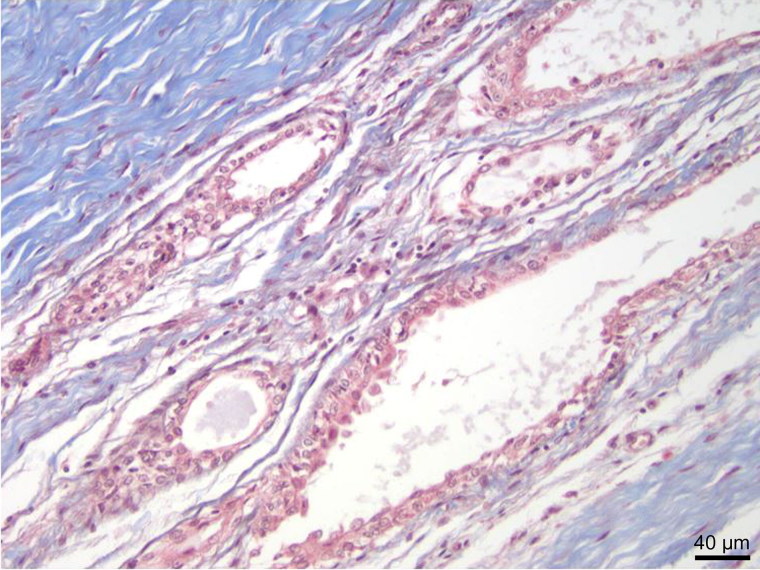
High power view of glands showing microvilli within glandular cells at bottom of image (Masson trichrome, ×400).

**Figure 5.**
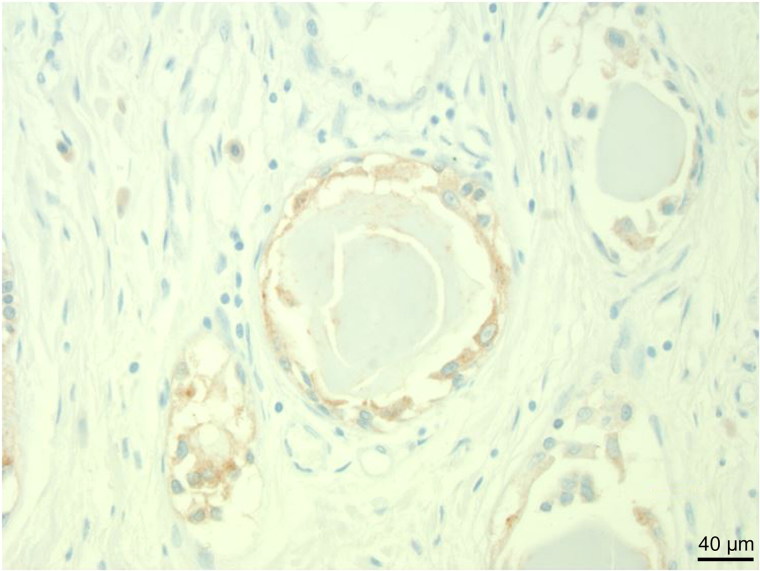
High power image of tumour cells staining positively for carcinoembryonic antigen (immunohistochemistry for carcinoembryonic antigen, ×400).

Pertinent autopsy findings included obesity and a 560 g heart. Her drug screen was negative. Because of the history of complete heart block, the cardiac conduction system was studied. This revealed replacement of the atrioventricular node (AVN) by glands lined by a single or double layer of cuboidal cells as well as solid nests of cuboidal cells. Some of the glands had intraluminal eosinophilic material and microvilli. They extended into, but did not replace, the His bundle (Figures 1–5) and extended into the atrial septum. This lesion was diagnosed as a CTAVN.

## Discussion

CTAVN can present with a range of symptoms, from none to SCD, as in this case. Typical symptoms include palpitations, dyspnoea, syncope and dizziness [5–7] and partial or complete heart block [6, 8–10]. A patient presenting with syncopal episodes followed by seizures has been misdiagnosed with epilepsy [7]. Many patients [4–11], including ours, have died despite pacemaker placement. CTAVN may be found incidentally, as described by Sharma et al. [12] (in explanted heart for postpartum cardiomyopathy), Suzuki et al. [13] (on thoracic CT scan), Law et al. [6] (epicardial pacemaker lead placement and repair of atrial septal defect, one case each) and Suárez-Mier et al. [14] (incidental findings in autopsies of accident victims).

Grossly, as in our case, CTAVN may be inapparent, but if visible grossly, the tumours have ranged from 0.5 mm to 3.0 mm, are yellow [13], cystic and may contain pultaceous debris [6]. Microscopically, the tumour cells can be squamous, cuboidal or transitional, and may be arranged in solid nests or glands. Glandular cells are connected with desmosomes and have short villi [6].

CTAVN have a 3:1 male to female preponderance, have a mean age of presentation of 38 years, with a range from newborn to 86 years. To date, eight cases have been diagnosed antemortem, all in women [13]. Diagnostic modalities have included transthoracic and transesophageal echocardiograms [5, 6, 15, 16], magnetic resonance imaging [5, 6, 13] and CT [13]. Initially asymptomatic tumours may become manifest later in life as the tumours grow in size due to glandular secretions [6].

The cells of CTAVN originate from endoderm as demonstrated by immunohistochemistry, staining positively for such endodermal markers as cytokeratin, epithelial membrane antigen (EMA), carcinoembryonic antigen (CEA), B72.3 [6, 17] and not staining for mesothelial markers [6, 17]. It is likely that when the developing heart is tubular and adjacent to the pharyngeal floor and the ultimobranchial body that endodermal tissue becomes incorporated into the heart, displacing or replacing the AVN [8, 11, 17, 18].

The fatal arrhythmia, despite pacemaker placement, may be ventricular tachycardia or ventricular fibrillation [9–11]. Ottaviani and Buja [1] believe the mechanism of death in CTAVN to be impaired electrical impulse propagation through abnormal atrioventricular junctional conducting tissue, which can lead to ectopic foci causing arrhythmias. CTAVN have been found in association with Wolf-Parkinson-White syndrome [6, 19], suggesting a role for CTAVN in pre-excitation.
